# Polypoid Cystitis and Bilateral Hydronephrosis Mimicking Urothelial Carcinoma

**DOI:** 10.1089/cren.2018.0098

**Published:** 2019-05-30

**Authors:** Jia-Lun Kwok, Molly Eng

**Affiliations:** Department of Urology, Khoo Teck Puat Hospital, National Healthcare Group, Singapore, Singapore.

**Keywords:** infection, polypoid cystitis, obstruction, ureteritis

## Abstract

***Background:*** Polypoid cystitis is infrequently seen in noncatheterized patients, occasionally posing challenging treatment options. We present a rare case of polypoid cystitis with bilateral upper tract obstruction mimicking a bladder tumor, needing staged endoscopic management.

***Case Presentation:*** A 45-year-old Asian man with a bladder tumor presented with lower urinary tract symptoms. Subsequent transurethral resection of bladder tumor histology revealed polypoid cystitis. This had caused intramural edema and obstruction of the distal ureters that were managed with initial local resection, ureteral dilatation with a period of ureteral stenting, and oral steroids. Subsequent intravenous urogram and diuretic renogram showed resolution of obstruction and resolution of hydronephrosis. He also had mixed lower urinary tract symptoms and hematuria managed with combination of solifenacin, finasteride, and tamsulosin.

***Conclusion:*** Polypoid cystitis with ureteral obstruction needs to be considered in differentials of a bladder tumor, and management may include a combination of endoscopic resection, ureteral stenting, and in resistant cases oral steroids can be considered to aid resolution of inflammation in the absence of infection.

## Introduction and Background

Polypoid cystitis is seen when benign urothelial mucosa assumes papillary and polypoid architectures secondary to inflammation and edema in the lamina propria in an inflamed bladder, with grossly noted polypoid or papillary lesions.^1^ It is infrequently seen without a history of catheterization, with most seen and more severe with repeated bladder catheterization.^2^ It can present as a mass on imaging and endoscopy, mimicking papillary urothelial neoplasms, and is preoperatively often misdiagnosed as bladder carcinoma.^3^ We report a case of polypoid cystitis with bilateral ureteral obstruction without a history of catheterization, and its management.

## Presentation of Case

Our patient is a 45-year-old Asian man with medical history of hypertension, and no other significant urologic history nor instrumentation. He is a nonsmoker. He presented with obstructive and storage urinary symptoms for 2 months. There was neither gross hematuria, dysuria, nor flank pain.

Physical examination was unremarkable. Digital rectal examination revealed a smooth 3.5 finger breadths prostate. Bladder ultrasonography measured a 32 cc prostate. Urine culture showed no growth. His creatinine was 102 μmol/L. He was started on tamsulosin 400 μg at night, but his symptoms persisted.

**FIG. 1.**
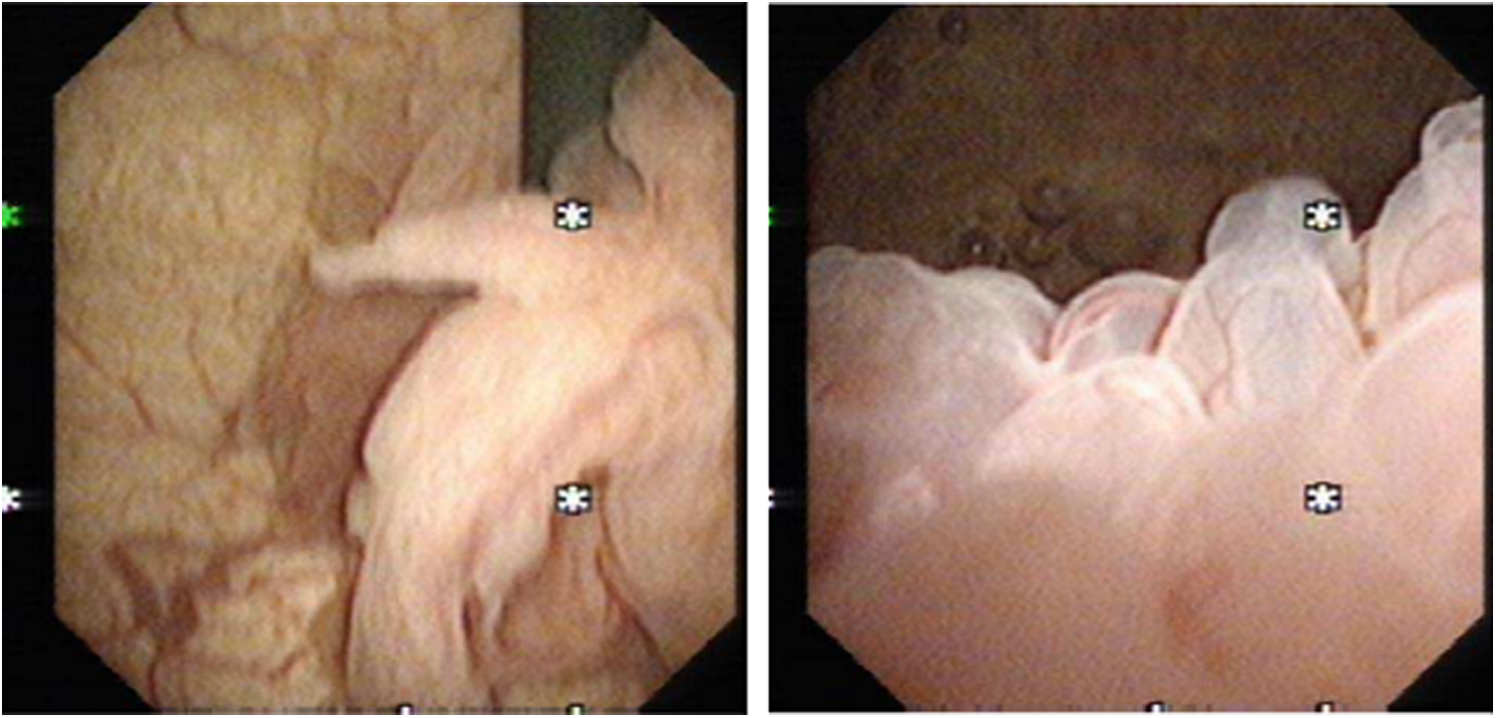
Flexible cystoscopy view of bladder tumor.

Flexible cystoscopy showed a large trigonal and bladder neck tumor >6 cm in size with possible extension to the prostate (Fig. 1). There were no urethral strictures, and the prostate had bilobar enlargement. Urine cytology revealed atypical cells suggestive of a low-grade urothelial neoplasm.

**FIG. 2.**
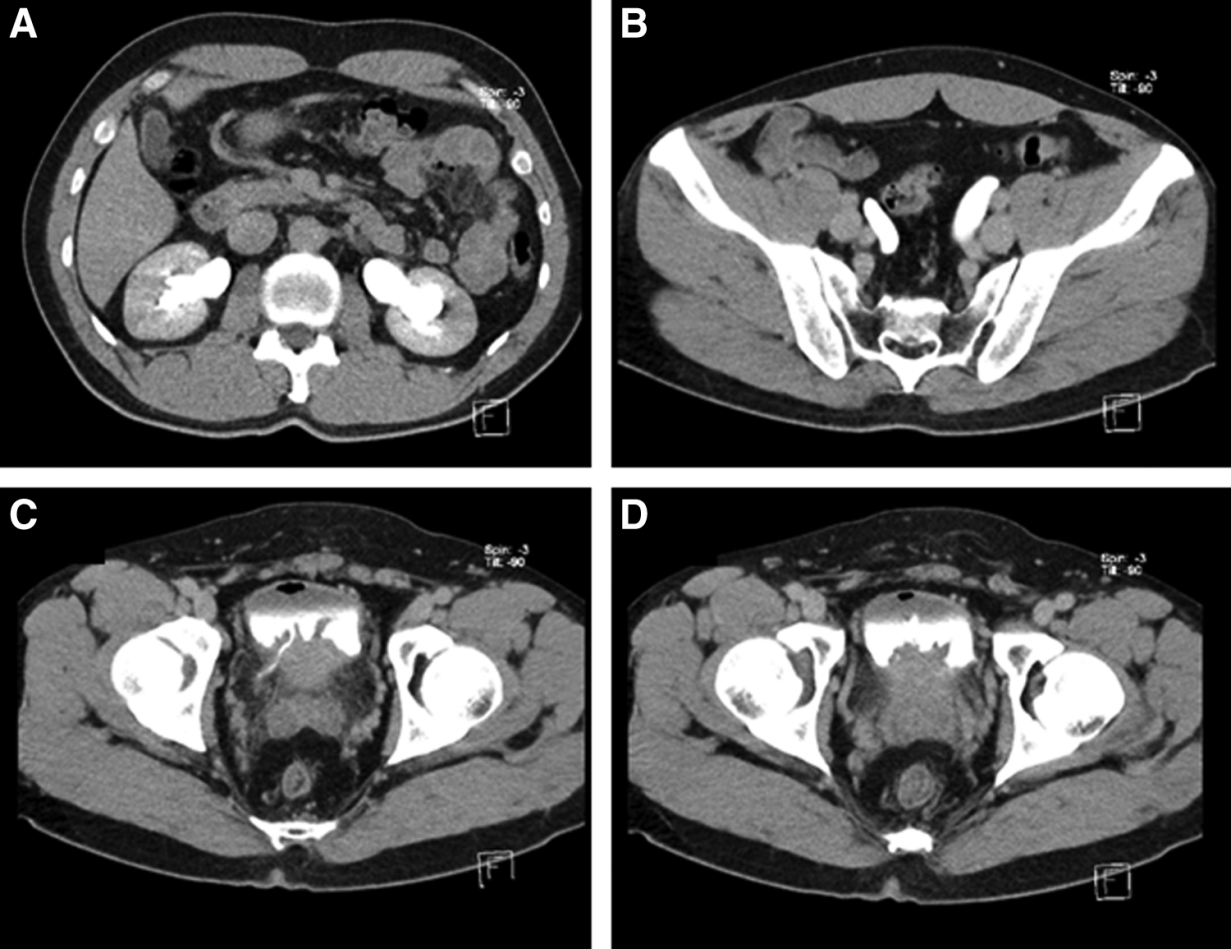
CT urogram delayed phase. **(A)** Bilateral hydronephrosis. **(B)** Dilated bilateral distal ureters. **(C)** Vesicoureteral junction involvement. **(D)** Bladder tumor at trigone.

CT urogram showed an irregular tumor arising from the posterior wall and base of the urinary bladder with vesicoureteral junction involvement, causing bilateral moderate hydroureteronephrosis (Fig. 2).

He underwent transurethral resection of the bladder tumor. The polypoid tumor was noted to be >6 cm in size, occupying the trigone and extending to the bladder neck with bilateral ureteral orifices not visible. Complete resection of the bladder tumor was achieved down to its base. Care was taken to avoid slow cutting strokes and only selective coagulation near the expected areas of the ureteral orifices. Prostatic stroma biopsies were taken at 5 to 7 o'clock from bladder neck to verumontanum and sent separately for histology. Random bladder biopsies were also taken.

Tumor histology returned as polypoid cystitis with features of prominent Brunn's nests with overlying epithelium showing reactive changes and scattered chronic inflammation, with detrusor muscle seen. No definite evidence of metaplasia, atypia, or malignancy was seen, confirmed by immunostaining. Prostatic stroma and random biopsy histology were benign.

Our patient returned with gross hematuria 1 month subsequently. Repeat CT urography showed stable bilateral mild hydronephrosis and dilation of both ureters up to the level of the ureteral orifices with short segment mural enhancement in the distal right ureter. Rigid cystoscopy showed the previous resection region in the trigonal area still inflamed. Retrograde pyelograms showed bilateral mild hydronephrosis with no filling defect, and both distal ureters showed areas concerning for obstruction. Bilateral 6F multilength ureteral stents (Contour™; Boston Scientific, MA) were, therefore, inserted.

Relook cystoscopy and ureteroscopy were performed 6 weeks later with findings of persistent inflammatory changes at the bladder trigone. Semirigid ureteroscopy found bilateral rigid and tight distal ureters. The ureteral mucosa was pink but edematous. There were no ureteral lesions up to the right upper ureter and left mid ureter; however, turbid urine was noted in the left ureter. Left ureteral urine was obtained and was negative on culture. Sequential dilatation of both ureters was performed with ureteral dilators (Cook Medical, IN) from 6F up to 9F with minimal resistance. In view of significant stent symptoms prior, bilateral 6F 24 cm dual durometer ureteral stents with bladder loops (Polaris™ Loop; Boston Scientific) were placed in this occasion.

**FIG. 3.**
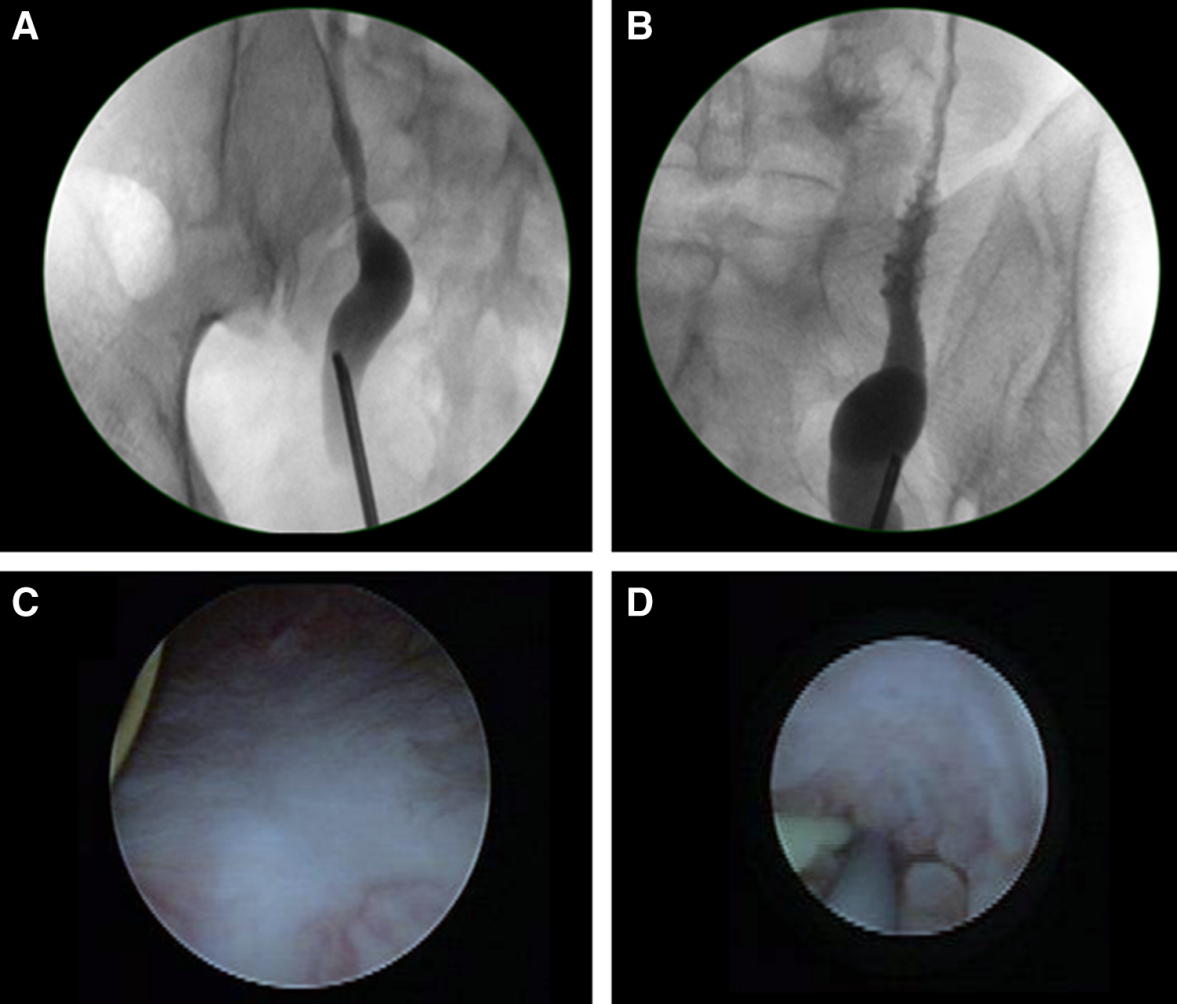
Endoscopic evaluation postureteral stenting. **(A)** Right capacious distal ureter. **(B)** Left ureteritis. **(C)** Healed bladder resection site. **(D)** Left proximal ureteritis—ureteroscopic view.

Decision was made to try him on a period of ureteral stenting for 3 months. Repeat rigid cystoscopy then showed resolution of inflammatory changes in the bladder mucosa. Bilateral retrograde pyelogram showed irregular outlines of the mid and upper ureter worse on the left, suggestive of ureteritis, with both distal ureters appearing capacious, and resolution of bilateral hydronephrosis. Left ureteroscopy to the left pelviureteral junction confirmed ureteritis changes seen over the upper and mid ureter, with small soft stones seen, which were removed (Fig. 3). The intramural ureters still felt rigid and edematous. In view of persistent delay in contrast emptying postretrograde pyelogram and some hold up at distal ureters, new bilateral 6F 24 cm dual durometer ureteral stents with bladder loops (Polaris Loop; Boston Scientific) were reinserted and oral steroids were considered for next line of treatment.

Our patient was then started on oral prednisolone 10 mg once a day for 1 month. After 2 weeks, both ureteral stents were removed through flexible cystoscopy. Oral prednisolone was tapered down to 5 mg once a day for another 3 months.

**FIG. 4.**
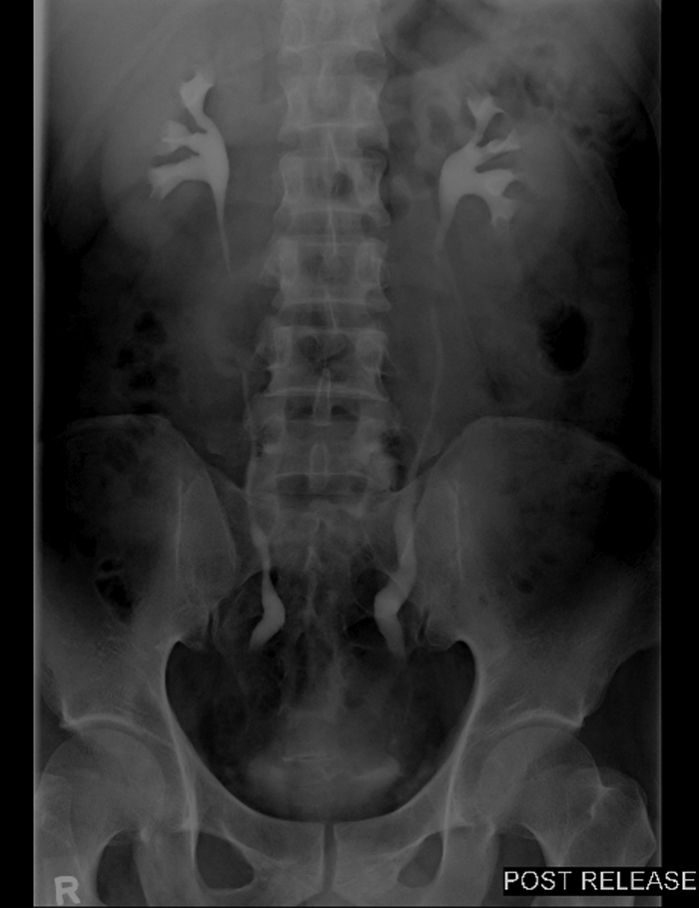
Intravenous urogram-stable patulous distal ureters with resolution of hydronephrosis.

Follow-up in 3 months with an intravenous urogram showed no hold up of contrast or filling defects and no hydronephrosis bilaterally, and oral prednisolone was tapered off. Repeat intravenous urogram 3 months later showed contrast hold up with mild dilation of bilateral distal ureters with no hydronephrosis (Fig. 4). A mercaptoacetyltriglycine renogram was done with equal differential renal function and no significant outflow tract obstruction. There was tracer stasis in both ureters followed by progressive clearance.

Our patient had persistent severe mixed lower urinary tract symptoms throughout the course of his treatment. Trial of nonsteroidal anti-inflammatory medications with short course etoricoxib 90 mg once daily and empiric oral antibiotics did not help improve his symptoms. Finasteride was started in view of persistent mixed lower urinary tract symptoms resistant to a 4-month trial of tamsulosin, in line with clinical findings of an enlarged prostate. His symptoms improved with a combination of finasteride 5 mg at night, tamsulosin 400 μg at night, and solifenacin 5 mg at night. His storage symptoms worsened with the use of double coiled ureteral stents and improved with the use of distal soft bladder loop stents. His lower urinary tract symptoms further improved greatly and were least with the use of oral prednisolone.

All urine cultures throughout his presentation and treatment were negative despite the extensive appearance of his polypoid cystitis and ureteritis.

Repeat intravenous urogram 15 months postremoval of bilateral ureteral stents showed stable bilateral marginally patulous ureters with no proximal ureteral dilation or hydronephrosis. On last review 27 months postremoval of bilateral ureteral stents, ultrasonography of the kidneys shows no hydronephrosis. Creatinine remains at his baseline. He remains well and is no longer on regular tamsulosin, finasteride, nor solifenacin.

## Discussion and Literature Review

Polypoid cystitis is a reversible inflammatory exophytic lesion of the bladder mucosa. Histologically there is normal or mildly hyperplastic urothelium, overlying a congested, chronically inflamed, and markedly edematous stroma. Metaplasia is rarely found.^1^ Microscopically, polypoid cystitis has broad-based polypoid excrescences.^2^ Edema inflammation with gross fronds of reactive exophytic structures is characteristic.^1^

What is unique in our patient is that of secondary bilateral hydronephrosis because of polypoid cystitis causing compression of the distal ureter. Prior reports had patients with hydronephrosis attributed to bladder outlet obstruction from prostate enlargement in benign prostatic hyperplasia.^2^ The pathophysiology of the ureteral obstruction is likely because of bladder mucosal edema and inflammation causing compression around the intramural part of the distal ureters, resulting in mechanical obstruction as bilateral hydronephrosis was already evident on CT before the first resection. The initial transurethral resection was mainly trigonal in location, with due care during resection ensuring only selective coagulation around areas overlying the expected position of both ureteral orifices. Subsequent ureteroscopy showed tight distal ureters with evidence of persistent inflammation along the intramural part of both ureters and, therefore, obstruction was felt to be more in keeping with the inflammatory process rather than strictures from previous resections.

Our patient found the use of a ureteral stent with a soft loop bladder end in an already inflamed bladder, giving less storage and symptoms bladder with less hematuria. There have been no prior case reports on the role of ureteral dilation, ureteral stenting, and types of ureteral stents used in mucosal edema secondary to polypoid cystitis.

Polypoid cystitis is usually reported in patients with a history of catheterization and located over the anterior and posterior wall of the bladder where the tip of the catheter sits.^1^ Our patient had neither catheterization nor urinary instrumentation prior and the polypoid cystitis was also located on the trigone and bladder neck.

Polypoid cystitis is also associated with colovesical fistulas, calculi, and history of radiation therapy.^3^ Our patient has none of the risk factors prior reported. Alike prior case reports, the initial suspicion in this patient was that of bladder carcinoma in view of the cystoscopic appearance of the lesion. Similarly, the urine cultures were consistently negative from this patient.^1^ Our follow-up was 36 months postdiagnosis, longer than what was previously reported.^1^

The usual reported management of polypoid cystitis is that of surgical resection and subsequent resolution,^4^ but concurrent medical therapy and treatment of upper tract obstruction are not well documented, including the role of corticosteroids in decreasing inflammation. Our patient did not have resolution of the cystitis or ureteral obstruction postresection, therefore, making the case unique.

Steroid use is still not established and is not standard treatment for noninfective cystitis. There is greater amount of literature on steroid use reported in treatment of cystitis glandularis, with a case report^5^ using a regime of 6 months of oral prednisolone (once daily dose of 10 mg oral for 3 months, then prednisolone 5 mg for 3 months). This use of steroids was extrapolated to our patient with polypoid cystitis. Multiple urine cultures were ensured to be negative before steroid therapy. Steroid use has also been reported to relieve obstruction in primary ureteritis causing ureteral obstruction.^6^ The decision for use of steroids was based on persistency of his lower urinary tract symptoms and persistency of inflammatory process despite resection, therefore, warranting a trial of steroid therapy. This was done after literature review of prior case reports and close discussion with the patient regarding rarity of the condition. From this case report, we propose considering the use of steroid treatment as a last resort for resistant cases of polypoid cystitis with persistent inflammation that does not resolve with conventional management.

## Conclusion

Polypoid cystitis should be considered as a differential in a bladder mass, and screening for upper tract obstruction should be done. There is a potential role of corticosteroids in decreasing symptoms and decreasing inflammation in polypoid cystitis with upper tract obstruction, in combination with endoscopic resection, ureteral dilation, and period of ureteral stenting.

## Abbreviation Used

CT: computed tomography
